# Infant processed food consumption and their interaction to breastfeeding and growth in children up to six months old

**DOI:** 10.1186/s12889-021-11539-5

**Published:** 2021-08-05

**Authors:** Renata Oliveira Neves, Luciano Santos Pinto Guimarães, Vera Lúcia Bosa, Leandro Meirelles Nunes, Clécio Homrich da Silva, Marcelo Zubaran Goldani, Juliana Rombaldi Bernardi

**Affiliations:** 1 Graduate Program in Child and Adolescent Health, Universidade Federal do Rio Grande do Sul, Porto Alegre, Brazil; 2 Biostatistics Unit, Research, and Graduate Studies, Hospital de Clínicas de Porto Alegre, Porto Alegre, Brazil; 3 Graduate Program in Food, Nutrition, and Health, Universidade Federal do Rio Grande do Sul, Porto Alegre, Brazil; 4 Department of Nutrition, Universidade Federal do Rio Grande do Sul, Porto Alegre, Brazil; 5 Department of Pediatrics, Universidade Federal do Rio Grande do Sul, Porto Alegre, Brazil

**Keywords:** Child nutrition, Breastfeeding, Complementary feeding, Processed food, Growth, Longitudinal studies

## Abstract

**Background:**

Evidences suggest that early processed food (PF) consumption may cause harm to infant health. During the first 6 months of life, it is not known whether the timing and quantity of this food group can impact breastfeeding and growth. The aim of the study was to analyze the associations between time of introduction and quantity of infant PF consumption with duration of breastfeeding and infant growth at 6 months of age.

**Methods:**

Data were longitudinally collected in six interviews, from birth to 6 months, in a sample of Brazilian newborns with adverse intrauterine environments. PF consumption was calculated by gravity score of processed foods (GSPF) in relation to feeding supply quality and time. For the analysis, the scores were divided into tertiles, making scores severities: Null, Mild, Moderate, and Severe. The interaction between GSPF and breastfeeding (exclusive and non-exclusive) and growth parameters (analyzed in Z-scores, by weight for height, weight for age, and body mass index for age) was tested.

**Results:**

A total of 236 infants were included in the study. Greater GSPF were associated with better rates of breastfeeding practices and higher growth indicators scores in the sixth month of infants. These findings were confirmed after adjustment for family income, maternal age, pre-gestational body mass index, and growth z scores at birth.

**Conclusion:**

The harms of eating PF in relation to breastfeeding and infant growth are more evident the greater and earlier they are consumed. Future studies should explore interventions to reduce and delay the consumption of these foods to prevent adverse health outcomes in later life.

**Supplementary Information:**

The online version contains supplementary material available at 10.1186/s12889-021-11539-5.

## Background

Nutritionally adequate feeding in the first year of life is critical for satisfactory growth and development of the child [1]. Inadequate nutrition, especially in childhood, can cause important repercussions to an individual’s health throughout the course of life and is intrinsically linked to cognitive and social development [2]. It was evidenced that the best feeding practice for infants during their first months of life is breastfeeding (BF) [3], since it provides several benefits to the infant, like protecting against allergic and respiratory diseases [4] and preventing the development of type 2 diabetes mellitus (DM) and obesity at short- and long-term [5], among others.

It is known that, after the sixth month of life, complementary feeding (CF) is necessary for both nutritional and developmental factors and they are an important transition from milk supply to family feeding [6]. There are large changes in diet during this period, with exposure to new foods, tastes, textures, and feeding experiences [6–8], and the duration of exclusive breastfeeding (EBF) could be associated with CF. It can be seen that solid food introduction at the right time, considered between 4 and 6 months of age, is correlated to EBF in the first month of life [9]. Studies have shown, in the same way, that duration of EBF for 4 months is related to higher daily consumption of vegetables, and EBF for less than 4 months is associated with lower consumption of fruits and vegetables and higher consumption of ultra-processed foods [10].

Children at around 6 months of age, especially in societies undergoing nutritional transition, generally receive a low-quality CF, with high consumption of ultra-processed foods and sweetened drinks [11]. Inappropriate infant feeding practices, such as early interruption of BF, the unsuitable introduction of CF and excessive consumption of industrialized products high amounts of sugar, fat, and sodium, can lead to feeding inadequacies throughout childhood [12].

Food processing contributes to food security, by ensuring that sufficient food is available for the population, and to nutrition security, by ensuring that its quality meets human nutrient needs. However, its excessive consumption contributes to the diet in a negative way, offering an unrestricted amount of energy, saturated fat, sugar, and sodium [13]. Among the processed foods most consumed by children under 6 months of age are gelatin and juice from cartons; followed by filled biscuits and salty snacks at the subsequent months of age [14].

Several factors may affect the infant nutritional development, such as the intrauterine environment. Fetus exposure to different intrauterine environments, such as DM [5, 15, 16], hypertensive disorders (HD) [17], intrauterine growth restriction (IUGR) [18], and smoking [19] may affect, posteriorly, BF practices, the offer of other kinds of milk, introduction of CF and child growth, by influencing birth weight and infant weight gain over the months.

It is not well known whether depending on the time of introduction and quantity of processed foods (PF) offered, they may act differently in health or breastfeeding practices, being more or less harmful to children in early childhood. Therefore, the aim of this study is to analyze the associations between time of introduction and quantity of infant processed food consumption with duration of breastfeeding and infant growth at 6 months old.

## Methods

### Participants

This is a longitudinal observational study performed with a convenience sample of mother-child pairs, selected between September 2011 to December 2016. The study is part of a larger prospective controlled project entitled Impact of Perinatal Environment Variations on the Health of the Newborn in the First Six Months of Life (IVAPSA) study. Further details about the IVAPSA study [20], as well as some baseline results [21] were previously published.

**Fig. 1:**
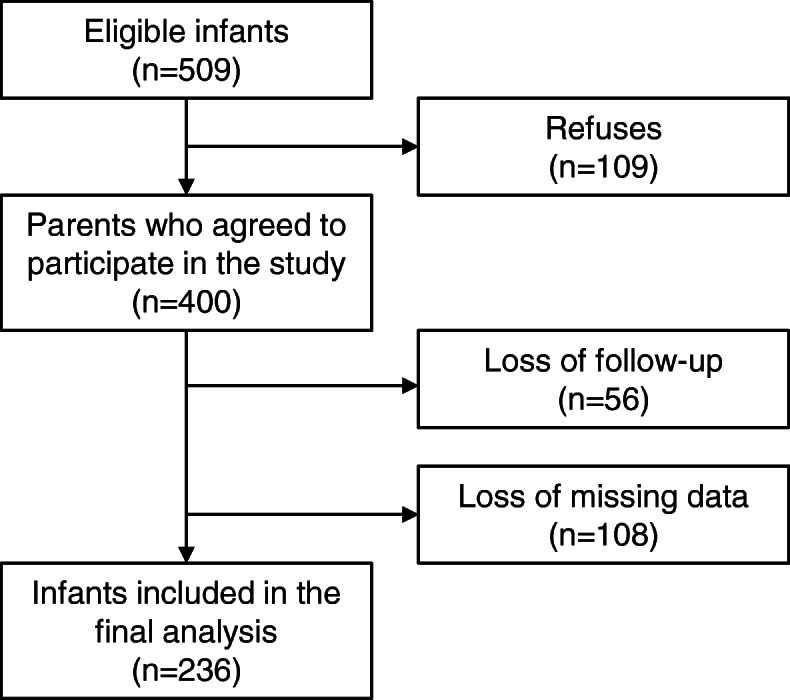
Infant selection flowchart, IVAPSA sample, Porto Alegre, 2011–2016

According to the IVAPSA study, the final sample size estimated was 521mother-child pairs, being 87 for each group of intrauterine environments and 174 pairs for the control group. The sample size calculation was described in detail in a previous publication [20]. Newborns were recruited at three hospitals in the city of Porto Alegre: Hospital de Clínicas de Porto Alegre (HCPA), Hospital Fêmina and Hospital Nossa Senhora da Conceição, the latter two belonging to the Grupo Hospitalar Conceição (GHC) (Fig. 1).

The infants were randomly selected based on hospital medical records. The data collection was carried out with interviews at 6 different times after delivery; 24–48 h, 7 days, 1 month, 3 months, and 6 months. These interviews occurred in the infants’ home or the Clinical Research Center of HCPA. Inclusion criteria consisted of pairs attended in the hospitals and belonging to the area covered by Porto Alegre city, Rio Grande do Sul. Mothers with HIV (Human Immunodeficiency Virus) positive test, newborn twins, who had congenital diseases at birth, gestational age less than 37 weeks, those who required hospital admission, those with a birth weight below 500 g, and pairs who did not attend the six-month interview were excluded from the study.

The IVAPSA sample consisted of mother-child pairs who had different backgrounds of adverse intrauterine environments exposure, such as tobacco, determined by affirmative answer for smoking during pregnancy, regardless of the number of cigarettes used per day; DM, defined as gestational diabetes, type 1 DM or type 2 DM; HD, designated by hypertensive disorders during pregnancy, whether it was classified as preeclampsia and eclampsia, preeclampsia superimposed on chronic hypertension, chronic hypertension; control, represented by mothers who did not have hypertension or DM, as well as those who were non-smokers; and IUGR, characterized by mothers with term infants small for gestational age who were below the percentile 5, according to Alexander curve parameters [22], whose causes were not smoking, HD, or DM. Women with more than one background were excluded from the study. The questionnaires used in this study were specially developed for the IVAPSA research, and are available as supplementary material (Supplementary Files 1, 2, 3 and 4).

### Covariates

Covariates were collected in the first and sixth interviews, using structured questionnaires on maternal demographic and socioeconomic information, pregnancy, childbirth and the infant variables. Maternal data were age (in years), education (years of study) and total family income (in Reais – Brazilian currency) and the number of past pregnancies (prior live births). Gestational data consisted of pre-gestational body mass index (BMI), expressed in kilogram by stature squared, Kg/m^2^, and the group of gestational clinical conditions (DM, HD, tobacco, IUGR and control). Infant data included sex, growth z-scores at birth, the pacifier and bottle-feeding use at 6 months, as well as the timing of introduction of cow’s milk or formula milk (in days).

### Feeding practices

For infant feeding practices analysis, data were collected from the second to the sixth interview, referring to infant feeding at hospital discharge; time in minutes of initiation of BF at postpartum, questions related to BF (e.g., Did the child breastfeed on the first day of life? If don’t, what did he receive? What was the feeding at hospital discharge? Does your baby breastfeed? If don’t, when did you stop breastfeeding?), weaning and introduction of CF.

The recommendations of infant nutrition analyzed were those proposed by “Feeding Guide for Brazilian Children Under Two Years” [23] and “Dietary Guidelines for the Brazilian Population” [24], both publications of the Ministry of Health of Brazil.

The BF variables were elaborated according to the World Health Organization (WHO) [25]: EBF, when the child received exclusively BF until 6 months, without any other liquid or solid, except for supplements and medicine and non-exclusive (non-EBF), when the child was breastfeeding, independent of other liquids or feeding offered. Then, a compute of the days of EBF-duration and non-EBF-duration was performed.

Foods consumed in the 24-h dietary recall, collected from the third to the sixth interview regarding all foods consumed in the day before, were transposed in Excel® version 2101 (Microsoft Office® Professional Plus pack), and categorized according to the degree of food processing (NOVA classification), proposed by “Dietary Guidelines for the Brazilian Population”, according to Monteiro and collaborators [26]:

- Natural or minimally processed foods: foods obtained directly from plants or animals, and may or may not be subjected to minimum changes;

- Culinary ingredients: products extracted from natural foods or directly from nature and used for seasoning, cooking, and create culinary preparations;

- Processed foods: products made essentially from the addition of salt or sugar to a natural or minimally processed food;

- Ultra-processed foods: products whose manufacturing involves various stages and processing techniques and various ingredients, many of them exclusively for industrial use.

The last 3 categories of processing (culinary ingredients, processed and ultra-processed foods) were grouped as “processed foods” (PF) for analyzes. After the compilation of the three groups of PF, a weighting was carried out by the numbers of items consumed, scoring more severity the earlier the consumption.

The gravity score of processed foods (GSPF) calculation occurred as follows: infants who consumed PF during the first 6 months of life were categorized according to the time of introduction of reported PF. To perform this categorization, a weighting was made by pointing each reported food item considered as PF with 10 points in the third interview, which represented the intake from the first 15 days of life; 7 points in the fourth interview, which represented the intake from 15 to 30 days of life; 5 points in the fifth interview, which represented the intake from 30 to 90 days of life; and 3 points in the sixth interview, which represented the intake from 90 to 180 days of life.

That is, the same food, if it was offered in more than one data collection, would be scored every time it was mentioned. Afterward, a sum of all points was performed and divided into tertiles, generating groups of mild severity (1–12 points), moderate (13–30 points), and severe (more than 30 points). Infants who did not consume any type of PF during the first 6 months of life, obtained null weighting.

### Child growth

Measurements of weight (in kilograms) and height (in centimeters) were collected at birth and in the 6-month interview. Child growth was assessed by WHO *Anthro*® software (Switzerland, 2005). The growth parameters were evaluated according to the anthropometric indices weight for height (WHZ), body mass index for age (BAZ), and weight for age (WAZ), proposed by WHO [27].

### Statistical analysis

Data were processed and analyzed with the statistical program SPSS®, version 18.0 (PASW Inc., Chicago, IL, EUA). Qualitative variables were expressed by absolute number and percentage, and Pearson’s chi-square test was used to detect differences between proportions. The Shapiro-Wilk test was used to test the normality of quantitative variables. Quantitative variables were expressed by the median values [interquartile range], and analyzed by the Kruskal-Wallis test with the Dunn Post Hoc test.

To compare the averages of growth variables between the different GSPF, it was performed an analysis of variance test (ANOVA), with the Tukey Post Hoc test. For asymmetric variables, the distribution of BF duration (exclusive and non-exclusive) was compared using the Kruskal-Wallis test; when significant, it was compared by Dunn Post Hoc test.

To perform the adjusted comparison for variables age, maternal pre-gestational BMI, income, and growth z-scores at birth, the BF time variables (exclusive and non-exclusive) were transformed into logarithms, correcting the values asymmetry. The comparison of growth variables and BF time logs were performed using the ANCOVA test. Results was presented by geometric mean and 95% confidence interval. For all analyzes, a significance level of 5% was considered.

### Ethical aspects

The mothers signed, in duplicate, the Free and Clarified Consent Term, at the time that preceded the first interview in the first 48 h after delivery. An identification number was defined for each child, maintaining the anonymity of the participants. The study was approved by the Research Ethics Committee of HCPA and GHC, under protocol numbers 11–0097 and 11–027, respectively.

## Results

A total of 236 infants were included in this sample. When comparing the characteristics from the included and excluded participants, most mothers from infants included in the sample were living with their partner (*p* = 0.047), had higher maternal education (*p* = 0.006), higher maternal age (*p* = 0.001), and a higher number of prenatal consultations (*p* = 0.004) (data not shown in tables).

Descriptive data according to the GSPF groups are shown in Table 1. Of the 236 infants, 28 were in the Null severity score group, 74 in the Mild severity score group, 66 in Moderate severity score group, and 68 were in the Severe group. The monthly family income of the Null group was significantly higher than the Moderate and Severe groups, as well as the Mild group in relation to the Severe group (*p* < 0.001). In the same way, the Null and Mild groups were differentiated by higher maternal education when compared to the other two scores groups (*p <* 0.001). Women of the Severe group were younger than the Null and Mild groups (*p* = 0.003). The Moderate group presented a significantly higher pre-gestational BMI than the Null group (*p* = 0.010). Regarding the different backgrounds of adverse intrauterine environments, controls showed a higher percentage in the Mild scores, when compared to the tobacco and DM groups. In contrast, the Severe group controls presented a lower percentage of participants compared to tobacco. In the Moderate severity, DM obtained a higher proportion in relation to HD (*p* = 0.013). The number of past pregnancies, the same as children-related variables, presented no significant differences between GSPF (*p* > 0.05).

**Table 1:** Characteristics of study participants according to severity scores of processed feeding, Porto Alegre, 2011–2016

Variables	Total (*n* = 236)	Gravity score food (GSF)				
		Null (*n* = 28)	Mild (*n* = 74)	Moderate (*n* = 66)	Severe (*n* = 68)	p
Family variables						
Family income (reais) * (median [25; 75])	1800 [1200; 2718]	2750 [1500; 3126]^a^	2.000 [1463; 2800]^ab^	1.500 [1072; 2200]^bc^	1.336 [0,976; 2313]^c^	< 0.001
Maternal education (years) (median [25; 75])	11.00 [8.00; 11.00]	11.00 [10.25; 12.37]^d^	11.00 [10.00; 11.00]^d^	9.00 [7.00; 11.00]^e^	8.50 [7.00; 11.00]^e^	< 0.001
Maternal age (years) (median [25; 75])	27.00 [21.00; 33.00]	29.00 [25.00; 34.75]^f^	28.50 [23.75; 33.00]^f^	26.00 [21.00; 33.25]^fg^	23.50 [19.00; 30,75]^g^	0.003
Pre-gestational BMI (Kg/m^2^) * (median [25; 75])	25.0 [21.6; 28.9]	22.52 [20.41; 26.82]^h^	25.04 [21.68; 28.16]^hi^	27.64 [22.97; 31.84]^i^	24.43 [21.71; 26.99]^hi^	0.010
Number of past pregnancies (n (%))						
Primiparous	92 (39.0)	15 (53.57)	30 (40.54)	28 (42.42)	19 (27.94)	0.094
Multiparous	144 (61.0)	13 (46.43)	44 (59.46)	38 (57.57)	49 (72.06)	
Adverse intrauterine environments (n (%))						
DM	49 (20.8)	7 (25.00)	10 (13.51)^A^	20 (30.30)^B^	12 (17.65)	0.013
HD	25 (10.6)	1 (3.57)	11 (14.86)	3 (4.55)	10 (14.71)	
Tobacco	43 (18.2)	7 (25.00)	6 (8.11)^A^	12 (18.18)	18 (26.47)^B^	
IUGR	25 (10.6)	3 (10.71)	8 (10.81)	5 (7.58)	9 (13.24)	
Control	94 (39.8)	10 (35.72)	39 (52.71)^B^	26 (39.39)	19 (27.93)^A^	
Infant variables						
Growth variables at birth (z-score) (mean (SD))						
WHZ	0.42 (1.09)	0.52 (1.17)	0.44 (0.90)	0.56 (1.12)	0.22 (1.22)	0.319
BAZ	0.15 (1.09)	0.26 (1.19)	0.22 (0.94)	0.28 (1.03)	−0.1 (1.22)	0.169
WAZ	−0.15 (1.06)	−0.11 (1.22)	−0.003 (0.96)	−0.12 (1.00)	−0.36 (1.12)	0.235
Cow’s milk introduction (days) (median [25; 75])	60 [17; 120]	27 [27; 27]	90 [22; 157]	90 [16; 133]	60 [16; 90]	0.109
Formula milk introduction (days) (median [25; 75])	22 [5; 81]	10 [1; 103]	15 [3; 90]	16 [4; 81]	30 [10; 75]	0.282
Sex distribution (n (%))						
Female	128 (54.2)	15 (53.6)	46 (62.2)	30 (45.5)	37 (54.4)	0.272
Male	108 (45.8)	13 (46.4)	28 (37.8)	36 (54.5)	31 (45.6)	
Pacifier at 6 months* (n (%))						
No	84 (35.9)	16 (57.14)	25 (34.25)	24 (36.92)	19 (27.94)	0.058
Yes	150 (64.1)	12 (42.86)	48 (65.75)	41 (63.08)	49 (72.06)	
Bottle feeding at 6 months* (n (%))						
No	20 (8.5)	5 (17.86)	8 (10.96)	3 (4.62)	4 (5.88)	0.136
Yes	214 (91.5)	23 (82.14)	65 (89.04)	62 (95.38)	64 (94.12)	

Subtitle: *BMI* body mass index, *DM* diabetes mellitus, *HD* hypertensive disorders, *IUGR* intrauterine growth restriction, *WHZ* weight for height z-score, *BAZ* BMI for age z-score, *WAZ* weight for age z-score

Different overwritten letters correspond to significantly different results

Statistical tests: chi-square of proportions, for qualitative variables, and Kruskal Wallis with Dunn Post Hoc, or ANOVA one-way, with Tukey Post Hoc test, for quantitative variables. Some variables, highlighted with an asterisk (*), may not totalize 236 participants, due to missing data

In the “adverse intrauterine environments” variable, the analysis of adjusted standardized residues smaller than 1.96 are overwritten with the capital letter A; and those greater than 1.96 are overwritten with the capital letter B

**Fig. 2:**
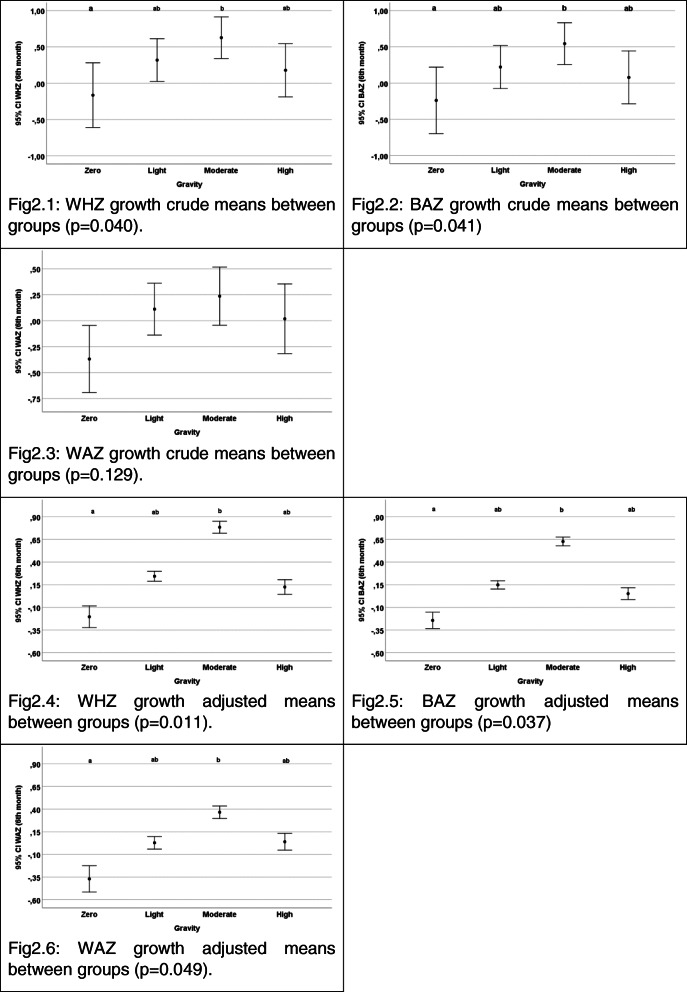
Crude and adjusted means distributions of growth, between different processed feeding severity scores, of IVAPSA sample included in the analysis, Porto Alegre, 2011–2016. Subtitle: WHZ = weight for height z-score; BAZ = BMI for age z-score; WAZ = weight for age z score. Adjusted means by total family income, maternal age, maternal pre-gestational BMI and baseline score of growth. Different letters represent categories with statistically different means

**Fig. 3:**
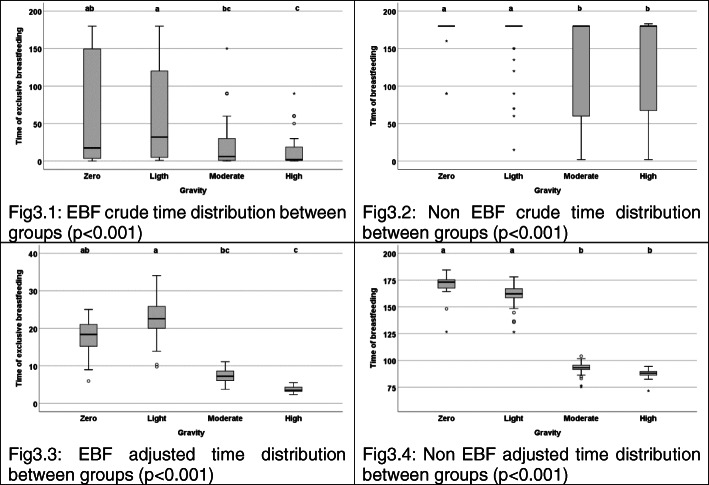
Crude and adjusted means distributions of duration of breastfeeding variables, between different processed feeding severity scores, of IVAPSA sample included in the analysis, Porto Alegre, 2011–2016. Subtitle: EBF = exclusive breastfeeding. Adjusted means by total family income, maternal age and maternal pre-gestational BMI. Different letters represent categories with statistically different means

In the crude analysis of growth and BF variables and their relationship with the GSPF (Table 2 and Figs. 2 and 3), it was perceived that the Moderate group obtained a higher WHZ, BAZ, and WAZ, compared to the Null severity (*p* = 0.040 and *p* = 0.041, respectively). Likewise, the Null and Mild scores demonstrated better rates at the BF variables. The duration of EBF was higher in the Null group in contrast to the Severe one, and in the Mild group compared to the Moderate and Severe (*p <* 0.001). Both the Null and Mild scores presented a greater general non EBF rate, in relation to the others (*p* < 0.001). The adjusted analysis for the total family income, maternal age, the maternal pre-gestational BMI, and growth z-scores at birth confirmed the crude findings. Relative to growth, the Null severity had a lower WHZ, BAZ and WAZ than the Moderate severity (*p* = 0.025 and *p* = 0.030, respectively). The findings of non-EBF also confirmed the crude analysis, in which the Null and Mild scores statistically differed from the Severe, and the Mild from Moderate, in the EBF duration (*p <* 0.001); while the Null and Mild severities demonstrated better rates of non-EBF, compared to the Moderate and Severe groups (*p <* 0.001).

**Table 2:** Crude and adjusted averages comparison of growth and breastfeeding duration variables, between different processed feeding severity scores of IVAPSA sample included on analysis, Porto Alegre, 2011–2016

Variables	Gravity score of processed food (GSPF)				
	Null (*n =* 28)	Mild (*n =* 74)	Moderate (*n =* 66)	Severe (*n =* 68)	P
Growth variables (in z-scores)					
Crude averages^I^ (mean (SD))					
WHZ	−0.16^a^ (1.15)	0.32^ab^ (1.24)	0.63^b^ (1.13)	0.18^ab^ (1.46)	0.040
BAZ	−0.24^c^ (1.18)	0.22^cd^ (1.25)	0.54^d^ (1.13)	0.08^cd^ (1.46)	0.041
WAZ	−0.37 (0.83)	0.11 (1.05)	0.24 (1.12)	0.02 (1.34)	0.129
Adjusted averages^III^ (mean (SD))					
WHZ	−0.28^e^ [− 0.84; 0.27]	0.21^ef^ [− 0.12; 0.54]	0.78^f^ [0.42; 1.13]	0.21^ef^ [− 0.16; 0.58]	0.011
BAZ	−0.29^g^ [− 0.83; 0.25]	0.12^gh^ [− 0.21; 0.46]	0.61^h^ [0.27; 0.96]	0.12^gh^ [− 0.25; 0.49]	0.037
WAZ	− 0.40^i^ [− 0.85; 0.06]	0.03^ij^ [− 0.31; 0.25]	0.36^j^ [0.07; 0.64]	0.16^ij^ [− 0.18; 0.43]	0.049
Breastfeeding variables (in days)					
Crude averages^II^ (median [25; 75])					
EBF time	17.50^kl^ [3.25; 149.75]	32.00^k^ [5.00; 120.00]	6.00^lm^ [1.00; 30.00]	2.00^m^ [1.00; 19.25]	< 0.001
Non EBF time	180.00^n^ [180.00; 180.00]	180.00^n^ [180.00; 180.00]	180.00^o^ [60.00; 180.00]	180.00^o^ [60.00; 180.00]	< 0.001
Adjusted averages^IV^ (mean (SD))					
EBF time^V^	19.20^pq^ [9.50; 39.10]	23.80^p^ [15.60; 36.30]	7.23^qr^ [4.68; 11.15]	3.27^r^ [2.07; 5.16]	< 0.001
Non EBF time^V^	175.40^s^ [120.70; 254.40]	162.60^s^ [129.10; 205.00]	91.60^t^ [72.60; 115.40]	86.70^t^ [67.30; 111.60]	< 0.001

Subtitle: I ANOVA one-way test, with Tukey Post Hoc test

II Kruskal-Wallis test, with Dunn Post Hoc test

III ANCOVA test, with Bonferroni Post Hoc test. Adjusted by total family income, maternal age, maternal pre-gestational BMI and baseline score of growth

IV ANCOVA test, with Bonferroni Post Hoc test. Adjusted by total family income, maternal age and maternal pre-gestational BMI

V Geometric means

Different letters represent categories with statistically different means

Breastfeeding duration variables were reported in days

## Discussion

The present study found that higher infant PF consumption, analyzed by GSPF, was associated with higher WHZ, BAZ, and WAZ, as well as lower EBF and non-EBF duration in the sixth month of infants. It should be emphasized that there was a meticulous division of feeding introduction, with regard to the degree of food processing, due to the fact that the literature shows that consumption of ultra-processed foods, as well as sweetened beverages, has their start early, in a frequent way, even before the age of 6 months [11].

In this survey sample, there were a large number of infants in Null, Moderate, and Severe scores, demonstrating the current food situation. As in other studies [28, 29], the early childhood eating practices were confirmed to be inadequate, compared to the recommendations for this age group [23], when replacing foods considered natural and healthy for the processed ones. The high consumption of PF by infants has shown to reflect a nutritionally poor diet with high amounts of carbohydrates, sugar, sodium, total and saturated fats, and energy density, and low amounts of proteins, fibers, vitamins, and minerals [30].

Researches have shown that the factors most strongly associated with this early and large PF consumption by infants are maternal education and age, family income, number of past pregnancies, BF in the first month of life, and the main caregiver not being the mother [28, 29]. In the present study, the sample descriptive characteristics corroborated with the literature, on what Null score, represented by infants that did not consume PF, had higher family income in comparison to Moderate (13–30 points) and Severe (more than 30 points) scores, as well as did Mild (1–12 points) score in relation to Severe. Maternal education and age were also higher in lower severity score groups. In contrast, women with Moderate scores presented higher pre-gestational BMI than Null ones. In the EDEN cohort, it was found that mothers who offer ultra-processed foods tend to be younger, with higher educational levels, and more likely to be obese [31].

Referring to adverse intrauterine environments, infants from the control group revealed greater number on the Mild severity, compared to tobacco and DM. In Moderate score, the infants from the diabetic group were in a higher proportion in relation to HD; and in Severe, the tobacco group predominated above control ones. In fact, these adverse intrauterine environments caused by different gestational clinical conditions had already been related to BF, by reducing the volume of milk produced and child response to breast milk in smoking [32, 33], leading to less BF duration in mothers who smoke, had gestational DM [34] and HD [35]; even as early introduction of CF, before 4 months of life [19]. However, relative to PF supply, studies are still scarce. A recent study observed that, in children exposed to gestational DM, BF only maintain its protection against childhood obesity if processed foods, like sugar-sweetened beverages intake, were also low [36].

Regarding child growth, in this research, the z scores were higher in Moderate severity score (13–30 points). However, these values did not exceed the normal range of eutrophic, and these data may not have great clinical relevance. A possible explanation would be that the period of child growth monitoring was considered short, being necessary more time so that greater differences could be noticed, being able, similarly, to relate to the PF; such as in pre-school age, which its consumption is a predictor of increased waist circumference in school-age [37].

Moreover, the consumption of PF, together with early introduction of wheat, cow’s or formula milk and shorter duration of BF interfere with diet quality, which may affect nutritional status at 2 years of age [38]. In the present study, the scores with the lowest PF intake demonstrated better rates of general and EBF and non-EBF duration, evidencing possible mutual protective effect.

Breast milk reduced the chances of consuming non-recommended foods, like cookies and crackers for children under 6 months old, industrialized yogurt between 6 and 12 months old, and soft drinks between 12 and 24 months of age [39]. Although surveys indicated the adverse effects of PF on daily basis, and government agencies strongly recommend not consuming them, it is known that, with globalization, it could be increasingly difficult to completely extinguish processed foods. On the other hand, while total exclusion is difficult to achieve, reducing consumption, with it not being part of the routine, can be a viable alternative for the population.

Furthermore, the study of the impact of ultra-processed foods on human health is essential, since they could impact the prevalence of autoimmune diseases, by inducing gut dysbiosis and promoting a pro-inflammatory response [40].

Among the strengths of the study can be highlighted the data collection in 6 interviews, in an important period of life, the first 6 months; making it possible to identify potential crucial periods for interventions. The analysis of feeding practices was performed meticulously and managed to encompass all aspects of infant feeding, by counting on a variety of collection instruments, increasing reliability of data, and reducing information bias given by the interviewees. Finally, the severity scores allowed an accurate analysis of PF consumption, providing different scores according to the moment and number of times offered. And also, pioneering research on infant feeding in the first 6 months considering, besides breastfeeding, the classification by type of food processing.

Instead, this study has shown limitations. The longitudinal character study generated a 41% loss of follow-up, decreasing the sample. This may have occurred due to the great housing mobility, characteristic of the most vulnerable social classes in Brazil; although, the analysis showed sufficient sample power, demonstrated by the significance of the main results. Because the sample has specific adverse intrauterine environments resulting from gestational clinical conditions, these results may not be generalized for the general population. Despite this, since this research used a sample of varied intrauterine environments with high prevalence, it is possible that the relations observed here may be interesting for the population of children of Brazil.

## Conclusion

The high and early consumption of processed foods by infants demonstrated a relationship with shorter breastfeeding duration and higher growth scores at their 6 months of life. Less supply from this food group, or its postponement, may reduce the harms of PF consumption at young ages. It is of great value, for a better understanding of the subject, that further studies be carried out with a longer period of follow-up.

## Supplementary Information

**Additional file 1: Supplementary File 1.** - IVAPSA Postpartum Questionnaire. Questionnaire applied 24–48 h after birth.

**Additional file 2: Supplementary File 2.** - IVAPSA 7-Days Questionnaire. Questionnaire applied 7 days after birth.

**Additional file 3: Supplementary File 3.** - IVAPSA 15, 30- and 90-Days Questionnaire. Questionnaires applied 15, 30 and 90 days after birth.

**Additional file 4: Supplementary File 4.** - IVAPSA 180-Days Questionnaire. Questionnaire applied 180 days after birth.

## Data Availability

The questionnaires used for the research are available as Supplemental File. Data can be requested from the responsible researcher, by email renataoliveiraneves@gmail.com.

## Abbreviations

BAZ: Body Mass Index for Age Z-Score
BF: Breastfeeding
BMI: Body Mass Index
CF: Complementary Feeding
DM: Diabetes Mellitus
EBF: Exclusive Breastfeeding
GHC: Grupo Hospitalar Conceição
GSPF: Gravity Score of Processed Food
HCPA: Hospital de Clínicas de Porto Alegre
HD: Hypertensive Disorders
IUGR: Intrauterine Growth Restriction
IVAPSA: Impact of Perinatal Environment Variations on the Health of the Newborn in the First Six Months of Life
PF: Processed Foods
WHO: World Health Organization
WAZ: Weight for Age Z-Score
WHZ: Weight for Height Z-Score
