# Supplementary material for: Infant processed food consumption and their interaction to breastfeeding and growth in children up to six months old
Source: BMC Public Health. 2021 Aug 5;21:1512. doi: 10.1186/s12889-021-11539-5 (PMC8340519; doi:10.1186/s12889-021-11539-5)
Supplement: Supplementary file 1 — Additional file 1: Supplementary File 1. - IVAPSA Postpartum Questionnaire. Questionnaire applied 24–48 h after birth. [file 12889_2021_11539_MOESM1_ESM.docx]

Supplementary File 1 – IVAPSA Postpartum Questionnaire

| Interview Date: __ __ / __ __ / __ __ |
| --- |
| Mother’s name: |
| GENERAL MOTHER DATA: |
| What is your birth date? __ __ / __ __ / __ __ |
| Have you ever got pregnant before?   1. No (1) Yes |
| If yes, number of children (including the current one): |
| What year of school did you study? |
| Last month, how much did the people living in your house earn? (include work income, benefits or retirement)  Income:  Person 1: R$ __ __ __ __ __ a month  Person 2: R$ __ __ __ __ __ a month  Person 3: R$ __ __ __ __ __ a month  Person 4: R$ __ __ __ __ __ a month  Person 5: R$ __ __ __ __ __ a month  TOTAL:________________ (77) Doesn’t know |
| Have you ever smoked or smoke tobacco cigarettes?  (0) No, never smoked (1) Yes, already smoked (2) Yes, currently smokes |
| GENERAL INFANT DATA: |
| Gender? (0) Female (1) Male |
| Date of birth? _______/_______/________ |
| Birth weight? ______________ grams |
| Birth lenght? _____________ cm |
| Did the child breastfeed on the first day of life? (0) No (1) Yes |
| If don’t, what did she received?  (0) Oral glucose solution (1) Intravenous glucose solution  (2) Formula 1st Semester (3) Other, which one?_____________________  (7) Don’t know (8) Not applicable |
| How many minutes after birth was the child breastfed for the first time?  ____________ minutes (5555) breastfed after 1st day (7777) Don’t know |
| What was the mother’s weight before getting pregnant?  ____________ kg (7777) Don’t know |
| Mother’s height: ______ cm (7777) Don’t know |
| SPECIFIC QUESTIONNAIRES |
| HYPERTENSIVE DISORDERS |
| What is the classification of your hypertension (medical record)?   1. Preeclampsia (2) Chronic hypertension (3) Eclampsia   (4) Preeclampsia superimposed by CH (5) Gestational hypertension (8) Not applicable |
| DIABETES |
| What is the classification of your diabetes (medical record)?  (1) DM type 1 (2) DM type 2 (3) Gestational Diabetes (GDM) |
