# Supplementary material for: Infant processed food consumption and their interaction to breastfeeding and growth in children up to six months old
Source: BMC Public Health. 2021 Aug 5;21:1512. doi: 10.1186/s12889-021-11539-5 (PMC8340519; doi:10.1186/s12889-021-11539-5)
Supplement: Supplementary file 2 — Additional file 2: Supplementary File 2. - IVAPSA 7-Days Questionnaire. Questionnaire applied 7 days after birth. [file 12889_2021_11539_MOESM2_ESM.docx]

Supplementary File 2 – IVAPSA 7-Days Questionnaire

| Interview Date: __ __ / __ __ / __ __ |
| --- |
| Feeding at hospital discharge:  ( 1 ) Exclusive breastfeeding  ( 2 ) Breastfeeding + infant formula  ( 3 ) Infant formula  ( 4 ) Other, which one? __________________________________________________ |
| Does your baby breastfeed?  (0) No (1) Yes |
| IF NO: |
| Why not? _______________________________________ (88) Not applicable |
| When did you stop breastfeeding? ____________ days (88) Not applicable |
| In addition to breast milk/formula/cow’s milk, do you offer any other food or liquid to your child?  (0) No (1) Yes |
| IF YES: |
| What foods/drinks are offered to the child?  Food 1: __________________________________________________  Food 2: __________________________________________________  Food 3: __________________________________________________  Food 4: __________________________________________________  Food 5: __________________________________________________ (88) Not applicable |
| When you introduced it, how many days old was the baby?  Food 1:_________days old  Food 2:_________ days old  Food 3:_________ days old  Food 4:_________ days old  Food 5:_________ days old (88) Not applicable |
| ANTHROPOMETRIC DATA: |
| Infant + mother’s weight  (1st) ________ kg (2nd) ________ kg Mean: ______ kg |
| Mother’s weight  (1st) ______ kg (2nd) ______ kg Mean: ______ kg |
| Baby length  (1st) ______ cm (2nd) ______ cm Mean: ______ cm |
