# Supplementary material for: Infant processed food consumption and their interaction to breastfeeding and growth in children up to six months old
Source: BMC Public Health. 2021 Aug 5;21:1512. doi: 10.1186/s12889-021-11539-5 (PMC8340519; doi:10.1186/s12889-021-11539-5)
Supplement: Supplementary file 3 — Additional file 3: Supplementary File 3. - IVAPSA 15, 30- and 90-Days Questionnaire. Questionnaires applied 15, 30 and 90 days after birth. [file 12889_2021_11539_MOESM3_ESM.docx]

Supplementary File 3 – IVAPSA 15, 30- and 90-Days Questionnaire

| Interview Date: __ __ / __ __ / __ __ |
| --- |
| Does your baby breastfeed?  (0) No (1) Yes |
| IF NO: |
| Why not? _______________________________________ (88) Not applicable |
| When did you stop breastfeeding? ____________ days (88) Not applicable |
| Does your baby receive or received pure water?  (0) No (1) Yes |
| IF YES: |
| When did you introduce?_________ days old. (88) Not Applicable |
| Does your baby receive or received tea?  (0) No (1) Yes |
| IF YES: |
| When did you introduce?_________ days old. (88) Not Applicable |
| Does your baby receive or received juice?  (0) No (1) Yes |
| IF YES: |
| When did you introduce?_________ days old. (88) Not Applicable |
| What type of juice is offered?  1. Natural (0) No (1) Yes  2. Concentrate – bottle or pulp (0) No (1) Yes (8) Not Applicable  3. Diluted (0) No (1) Yes  4. Artificial – powder/syrup (0) No (1) Yes |
| Does your baby receive or received soft drink?  (0) No (1) Yes |
| IF YES: |
| When did you introduce?_________ days old. (88) Not Applicable |
| Does your baby receive or received any milk other than breast milk?  (0) No (1) Yes |
| IF YES: |
| When did you introduce?_________ days old. (88) Not Applicable |
| What type of milk is offered?  1. Follow-up milk – NAN, Nestogeno, Milupa, Aptamil. (0) No (1) Yes  2. Whole powdered milk – Ninho, Glória, Elegê. (0) No (1) Yes  3. Cow’s milk (box or bag). (0) No (1) Yes  4. Special milk – Alfarré, Sobee, NAN Soy, Aptamil Soja, SoyMilk. (0) No (1) Yes  5. Another type of milk. Which one? ____________________________________(8) Not applicable |
| Are any other products added to milk?  (0) No (1) Yes |
| IF YES: |
| What products are used to thicken, dilute, enrich or sweeten milk?  1. Un enriched cereals (oats, corn starch) (0) No (1) Yes  2. Enriched cereals (Mucilon, Arrozina, Farinha Láctea) (0) No (1) Yes  3. Sugar (0) No (1) Yes  4. Chocolate powder (0) No (1) Yes  5. Oil (0) No (1) Yes  . 6. Water (0) No (1) Yes  7. Another type of product. Which one? _____________________________(8) Not applicable |
| When did you introduce?_________ days old. (88) Not Applicable |
| Does your baby eat other (solid) foods?  (0) No (1) Yes |
| ANTHROPOMETRIC DATA: |
| Infant + mother’s weight  (1st) ________ kg (2nd) ________ kg Mean: ______ kg |
| Mother’s weight  (1st) ______ kg (2nd) ______ kg Mean: ______ kg |
| Baby’s weight  (1st) ______ kg (2nd) ______ kg Mean: ______ kg |
| Baby’s length  (1st) ______ cm (2nd) ______ cm Mean: ______ cm |
| \| When did you introduce the following foods to your child? \| **No** \| **< 1st month** \| **1st month** \| **2nd month** \| **3rd month** \| **4th month** \| \| --- \| --- \| --- \| --- \| --- \| --- \| --- \| \| Additional sugar (milk, juice or tea) \|  \|  \|  \|  \|  \|  \| \| Powdered chocolate \|  \|  \|  \|  \|  \|  \| \| Honey \|  \|  \|  \|  \|  \|  \| \| Coffee \|  \|  \|  \|  \|  \|  \| \| Mashed fruit \|  \|  \|  \|  \|  \|  \| \| Salted baby food/soup \|  \|  \|  \|  \|  \|  \| \| Industrialized soup \|  \|  \|  \|  \|  \|  \| \| Vegetables \|  \|  \|  \|  \|  \|  \| \| Legumes (eg beans, lentils) \|  \|  \|  \|  \|  \|  \| \| Family food \|  \|  \|  \|  \|  \|  \| \| Meat (cow, chicken, pork, fish) \|  \|  \|  \|  \|  \|  \| \| Offal meat (eg liver, gizzards) \|  \|  \|  \|  \|  \|  \| \| Egg \|  \|  \|  \|  \|  \|  \| \| Sausages (eg ham, sausage, Bologna, salami) \|  \|  \|  \|  \|  \|  \| \| Wafer \|  \|  \|  \|  \|  \|  \| \| Cookie \|  \|  \|  \|  \|  \|  \| \| Danoninho (petit suisse cheese) \|  \|  \|  \|  \|  \|  \| \| Chocolate \|  \|  \|  \|  \|  \|  \| \| Candy or lollipop \|  \|  \|  \|  \|  \|  \| \| Salted Snack \|  \|  \|  \|  \|  \|  \| \| Gelatin/pudding \|  \|  \|  \|  \|  \|  \| \| Ice cream/iced popsicles \|  \|  \|  \|  \|  \|  \| \| Fried foods (eg fried potato, breaded chicken) \|  \|  \|  \|  \|  \|  \| |
|  |
